# Prevalence and predictors of MRSA carriage among employees in a non-outbreak setting: a cross-sectional study in an acute care hospital

**DOI:** 10.1186/s12995-019-0226-0

**Published:** 2019-03-12

**Authors:** Melanie Schubert, Daniel Kämpf, Lutz Jatzwauk, Franziska Kynast, Annette Stein, Ruth Strasser, Madeleine Dulon, Albert Nienhaus, Andreas Seidler

**Affiliations:** 1 Institute and Policlinic of Occupational and Social Medicine, Medical Faculty Carl Gustav Carus, Technische Universität Dresden, Dresden, Germany; 2 Department of Hospital Infection Control, Medical Faculty Carl Gustav Carus, Technische Universität Dresden, Dresden, Germany; 3 Heart Center, University Hospital of the Technical University Dresden, Dresden, Germany; 4 Medical Faculty Carl Gustav Carus, Technische Universität Dresden, Dresden, Germany; 5 Department of Occupational Medicine, Public Health and Hazardous Substances, Institution for Statutory Accident Insurance and Prevention in the Health and Welfare Services, Hamburg, Germany; 6 Competence Centre for Epidemiology and Health Services Research for Healthcare Professionals (CVcare), University Medical Centre Hamburg-Eppendorf (UKE), Hamburg, Germany

**Keywords:** Health care workers, MRSA prevalence, Non-outbreak setting, Risk factors, MRSA strain

## Abstract

**Background:**

Health care workers have an increased risk of being infected with Methicillin-resistant *Staphylococcus aureus* (MRSA), though little information is available about how prevalent (dormant) MRSA colonization is among health care workers. The aim of this study was to estimate the prevalence and predictors of MRSA carriage in a non-outbreak setting in a university hospital in Germany.

**Methods:**

The entire staff of a university hospital heart center for cardiologic maximum medical care and cardiac surgery were invited to participate in a cross-sectional study (*N* = 575). The sampled population included health care workers as well as employees with no close patient contact.

A questionnaire concerning personal and occupational risk factors as well as lifestyle and demographic factors was applied and nasal swabs were taken. In total 180 persons (31.3%) participated in the study.

**Results:**

The majority of study participants had close contact to patients at work (*n* = 149, 82.8%). Thereof, about one-third had contact to MRSA-patients (*n* = 53, 35.6%), and most reported wearing protective clothing (*n* = 44, 83.0%). None of the administrative staff tested positive for MRSA and only one in 149 persons (0.7%, CI 0.00–0.02) with close patient contact carried MRSA (strain *CC1-MRSA-IV*). This person had close contact to patients with MRSA, less than 1 year of work experience, and had been treated with antibiotics within the last 12 months.

**Conclusion:**

The results of our study suggest low point prevalence rates of MRSA colonization in health care workers in a non-outbreak setting.

## Background

*Staphylococcus aureus* is a ubiquitous gram-positive bacterium that most commonly colonizes the human nasal vestibule and skin. The methicillin-resistant form – Methicillin-resistant *Staphylococcus aureus, abbreviated* MRSA – is an important cause of nosocomial infections worldwide [1]. Though a general decrease in MRSA has been observed by the European Centre for Disease Prevention and Control for the European Union [2], MRSA still accounts for over 40% of healthcare-associated infections in the European Union [3]. Health care workers (HCWs) may serve as a reservoir and vehicle of spreading MRSA [4, 5]. A systematic review assessing 191 MRSA outbreaks found that HCWs were the source in 11 of 191 outbreaks, and asymptomatic carriers were the source in three of the outbreaks. Moreover, transmission of MRSA to household members is also likely to occur [6–8]. Routine screening of HCW staff is controversial, although it has been shown that screening could help decrease MRSA infection rates [9, 10].

Little is known about the prevalence of MRSA carriage in non-outbreak situations. In a systematic review including studies from non-outbreak settings in Europe and the US, the pooled prevalence of MRSA carriage in HCWs was 1.8% (95% CI 1.34–2.50), and increased to 4.4% (95% CI 3.98–4.88) when one study from the Netherlands was excluded from the analysis [11]. This latter prevalence is similar to the results of a recent study performed in a non-outbreak setting in nine German acute care hospitals which observed MRSA carriage prevalence rates for medical staff of 4.6% [12].

MRSA carriage rates have been shown to be highest in nursing staff. The aforementioned systematic review also found that nurses had an increased risk of 1.72 (95% CI 1.07–2.77) when compared with medical staff, and a risk of 2.58 (95% CI 1.83–3.66) when compared with other healthcare staff [11]. Also, in the recent German study by Sassmannshausen and colleagues [12] mentioned above, nurses had a higher MRSA carriage prevalence compared to physicians (5.6% versus 1.2%). Ibarra and colleagues found similar risks for physicians (13%), nurses (12%), and other healthcare professionals in the US [13]. Risk factors for MRSA carriage in HCW are a known history of MRSA infection or colonization, direct contact with patients with MRSA infections, recent hospitalization or emergency department visit, and recent antibiotic use [13–15]. Other risk factors are chronic skin disease, poor hygiene practices, and working in countries with endemic MRSA. Nurses with occupational skin disease have been shown to have a higher risk for MRSA colonization [16].

MRSA carriage might be chronic or intermittent, where persons are colonized for a short time period. One form of intermittent carriage is the transient carriage, where MRSA isolated after work is gone before next day’s duty [17]. MRSA eradication is usually successful in the majority of HCWs (88%) [15], and successful decolonization (with mupirocin) has been shown in 94% of cases 1 week after treatment [10]. About 5% of MRSA colonized HCWs develop clinical infections [15] which may progress into serious disease or have negative consequences at work [18, 19]. In Germany, about 50 potentially occupationally related cases involving Methicillin-resistant *Staphylococcus aureus* (MRSA) were reported per year in the last 5 years [20]. However, most of these cases concerned carriage of MRSA, which is not considered an occupational disease according to German regulations.

The present study aimed to describe the prevalence of MRSA carriage in a German acute care hospital and to identify risk factors associated with colonization. Results should provide a basis for improved workplace risk assessment identifying opportunities to prevent infections.

## Methods

The study was conducted from July 2014 to May 2015. All employees (*N* = 575) of the heart center (“Herzzentrum Dresden GmbH”), the specialized cardiology care and cardiac surgery center of the Technische Universität Dresden’s teaching hospital, were invited to participate. An anonymous invitation, including study information, a questionnaire and informed consent, was sent with the monthly pay slip to each employee. The study team also presented the study with public talks at the center. This was done to inform the employees about the study and to give employees the opportunity to ask questions. For study participation, employees were asked to sign the informed consent and complete the questionnaire. The questionnaire included standard questions on personal characteristics and work, as well as questions concerning occupational and personal risk factors for MRSA. MRSA risk factors considered were predominately derived from a review by Albrich and Harbarth [15], a questionnaire used by the German Social Accident Insurance Institution for the health and welfare services (Berufsgenossenschaft für Gesundheitsdienst und Wohlfahrtspflege – BGW) for staff in nursing homes [21], and a literature search.

For sampling, the study team was on-site at the Herzzentrum Dresden for 2 days. Participants were invited to the examination room provided by the center. Initially, the informed consent and the completed questionnaire were collected and participants had the opportunity to ask questions. Then, samples were collected by a trained member of the study team using a swab from the anterior nares, which are the main reservoir for MRSA [11]. Employees had the choice to either participate anonymously or to receive feedback concerning the findings of their MRSA-analysis (either via post or by collecting the result in person at our policlinic).

All nasal swab samples were analyzed at the Institute for Medical Microbiology and Hygiene, Medical Faculty Carl Gustav Carus of the Technische Universität Dresden according to the quality guidelines of the laboratory. Antibiogram-resistogram typing with VITEK 2 and a PBP2a-Culture Colony Test (Alere Technologies GmbH) was done for positive MRSA samples. Moreover, MRSA-positive samples were genotyped using a “*S. aureus* Genotyping Kit 2.0” (Alere Technologies GmbH).

Descriptive statistics are shown for all data. Due to low sample size no statistical testing was performed. However, we included 95% confidence intervals for MRSA colonization applying the “Rule of Three” [22]. Information for mean age of all employees and percentage of women working at the Herzzentrum Dresden was provided from the office of human resources. The mean age was 40.5 years, and 72% of employees were women.

In total, 180 employees participated in the study, resulting in a response of 31.3%. Only three persons participated anonymously in the study; the majority of participants received the feedback via post (*n* = 171). Six participants collected the results from the study team.

## Results

Descriptive statistics for personal characteristics, as well as work-related and private risk factors for MRSA are displayed in Table 1. In short, predominately women participated in the study (68.9%). The majority of participants was between 40 and 49 (31.1%) and 30–39 (24.4%) years old, lived in a partnership (75.0%), worked as a nurse (55.6%), had a university entrance diploma (52.2%) and 11–20 years of professional experience (41.1%).

**Table 1:** Descriptive statistics

	Number	Percent
Demography		
Sex (Proportion of women)	124	68.9
Age in years		
< 20	1	0.6
20–29	35	19.4
30–39	44	24.4
40–49	56	31.1
50–59 years	34	18.9
≥ 60 years	8	4.4
Missing	1	0.6
Education		
Secondary school graduation	81	45.0
High school, University entrance qualification	94	52.2
Other	4	2.2
Missing	1	0.6
Occupation		
Physician	19	10.6
Nurse	100	55.6
Therapist	7	3.9
Medical technical assistant	8	4.4
Administrative personnel	29	16.1
Others	16	8.9
Missing	1	0.6
Field of activity		
ICU/ IMC/ OP	70	38.9
Normal ward	54	30.0
Diagnostic	13	7.2
Administration/ technician	17	9.4
Others	25	13.9
Missing	1	0.8
Professional experience in years		
≤ 1	12	6.7
1–5	24	13.3
6–10	24	13.3
11–20	74	41.1
21–40	41	22.8
> 41	4	2.2
Missing	1	0.6
Partnership		
Yes	135	75.0
No	45	25.0
Household		
One-person	35	19.4
Multi-persons	144	80.0
Missing	1	0.6
Work-related MRSA-risk factors		
Having close contact to patients (washing, dressing changes,..)		
Yes	112	62.2
No	67	37.2
Missing	1	0.6
Having contact to MRSA-patients within the last 4 weeks		
Yes	53	29.4
No	66	36.7
Unknown	58	32.2
Missing	3	1.7
Wearing protective cloths when having contact to MRSA-patients		
Yes, always	44	83.0
Occasionally without	3	5.7
Missing	6	11.3
What kind of protective cloths when having contact to MRSA-patients		
Surgical face mask, disposable gloves and lab coat	42	79.2
Surgical face mask and disposable gloves	5	9.4
Surgical face mask	1	1.9
Disposable gloves	1	1.9
Not applicable	1	1.9
Missing	2	2.3
Working abroad		
Yes (India/Kazakhstan, Spain, USA)	3	1.7
No	164	91.1
Missing	13	7.2
Private MRSA-risk factors		
Caring for relatives at home		
Yes	9	5.0
No	171	95.0
Having contact to MRSA-carriers		
Yes	3	1.7
No	132	73.3
Unknown	45	25.0
Having contact to persons in need for care within the last 4 weeks		
Yes	22	12.2
No	158	87.8
Working in the ambulant sector outside work		
Yes	2	1.1
No	178	98.9
Having contact to farm animals		
Yes	9	5.0
No	171	195.0
Having contact to pets		
Yes	89	49.4
No	89	49.4
Missing	2	1.1
Chronic skin disease		
Yes, own diagnosis	4	2.2
Yes, doctor’s diagnosis	15	8.3
No	158	87.8
Missing	3	1.7
Chronic respiratory disease		
Yes, own diagnosis	7	3.9
Yes, doctor’s diagnosis	15	8.3
No	154	85.6
Missing	4	1.7
Diabetes mellitus		
Yes, own diagnosis	1	0.6
Yes, doctor’s diagnosis	3	1.7
No	167	92.8
Missing	9	5.0
Having had MRSA		
Yes	4	2.2
No	176	97.8
Use of antibiotics within the last12 months		
Yes	57	31.7
No	121	67.2
Missing	2	1.1
Hospital stay within the last 12 months		
Yes	16	8.9
No	161	89.4
Missing	3	1.7

About one third (*n* = 53) have had contact to MRSA patients within the last 4 weeks. A similar proportion of participants (*n* = 58) reported not knowing whether they had contact or not. Of the employees reporting contact with MRSA patients, 83% of participants reported wearing protective clothing and about 80% of these persons reported wearing a surgical face mask, disposable gloves, and a lab coat. Three persons reported that they had occasional contact without protective cloths and another six persons did not answer the question.

Only three participants had contact to MRSA carriers at home. Nine persons were caring for relatives and 22 had contact to persons in need for care within the last 4 weeks. About half of participants had contact to pets (*n* = 89) and only a few to farmed animals (*n* = 9).

Four participants had MRSA in the past. Chronic skin disease and chronic respiratory disease were reported by about 10% each. Few participants suffered from diabetes mellitus (doctor’s diagnosis: *n* = 3, own diagnosis: *n* = 1). About one third used antibiotics within the last 12 months (*n* = 57) and 16 participants had a hospital stay within the last 12 months.

None of administrative staff tested positive for MRSA. One of 149 persons with close patient contact carried MRSA (CI 0.00–0.02). This person worked on a normal ward (occupation: “others”) and had less than a year of work experience. This participant had close contact to MRSA patients within the last 4 weeks and reported wearing protective clothing (surgical face mask, disposable gloves and lab coat) at all times. Furthermore, the participant was not aware of having contact to MRSA carriers away from work, did not care for relatives, and had no contact to persons in need for care within the last 4 weeks. The positively-tested participant did not work in the ambulant sector outside of the heart center and had neither contact to pets nor to farm animals. The participant had no chronic disease, but was treated with antibiotics within the last 12 months.

Genotyping revealed the strain CC1-MRSA-IV (WA MRSA – 1/57). Spa types associated with this strain are t127, t386, t590, t922, t2601. The isolate was mecA positive and PVL negative. The sample tested positive for the leukotoxin gamma-hemolysin. Gene virulence factors were enterotoxin H *(she),* hemolysin gamma*/*leukocidin component B (*lukF),* hemolysin gamma*/*leucocidin component C (*lukS),* hemolysin gamma*/*leukocidin component C, allele from ST22 and ST45 (*lukS ST22 + ST45),* hemolysin gamma component A *(hlgA),* leukocidin component D (*lukD),* leukocidin component E (*lukE),* staphylokinase (*sak)* and staphylococcal complement inhibitor *(scn).* The sample tested negative for Panton-Valentine leukocidin F component (*lukF-PV)* and Panton-Valentine leukocidin S component (*lukS-PV).*

The isolate was multi-drug resistant: Penicillinase (beta-lactamase gene, *blaZ*), MLS-antibiotics (rRNA metyltransferase associated with macroscelide/ lincosamide resistance, *crm(C)*), aminoglycosides (aminoglycoside phosphotransferase (neo−/kanamycin), *aphA3*) and miscellaneous genes (streptothricin acetyltransferase, *sat*; tetracycline efflux protein, *tet(K)* and chloramphenicol/florfenicol exporter, *texA*).

## Discussion

Our results suggest a low prevalence of MRSA in HCWs of less than 1% (1 in 149 HCW). The observed MRSA carriage prevalence is lower than those reported by most other study groups of HCWs in German acute care hospitals, such as 4.6% [12], 5.3% [23], 4.0% [24], 3.2 and 2.8% [25]. This observation suggests a possible decline in MRSA carriage among HCWs in Germany. According to hygiene regulations at the heart center, workers with infectious diseases are prohibited from working with patients if a transmission of disease cannot be ruled out. On admission to the acute care hospital, patients are screened for MRSA (and 4 MRGN) if they have a history of MRSA, have chronic wounds, are on dialysis, or were transferred from other hospitals and rehabilitation centers. Patients with MRSA are isolated in single rooms. HCWs must wear gloves, a coat and a face mask when having direct contact with patients screening positive for MRSA. Successful decolonization of a patient is confirmed by three negative nose smears and one negative throat smear at least 3 days after antiseptic treatment. There were no MRSA outbreaks in the last 10 years at the hospital. A general decrease in MRSA has been observed by the European Centre for Disease Prevention and Control for the European Union [2]. Furthermore, well qualified and sufficient hygiene personnel are essential for preventing nosocomial infections. In Saxony, the number and qualification of hygienists in health facilities was legally determined in 2012 [26]. Thus, the low observed prevalence of MRSA might have resulted from sufficient hygiene staff education and compliance to hygiene measures [27].

The MRSA-colonized person worked on a normal ward (occupation: “others”) and had less than a year of work experience. The participant had close contact to patients with MRSA within the last 4 weeks but reported wearing protective clothing at all times. The person was also treated with antibiotics within the last 12 months. These are common risk factors for MRSA colonization in HCWs [13, 28]. Thus, although the sample size is too low to make general assumptions about risk factors for MRSA carriage, our results fit to the body of evidence for potential risk factors [28, 29].

The isolated strain was not one of the more common nosocomial strains in Germany. The isolate was CC1-MRSA-IV which is identical to WA MRSA-1/57 from Western Australia [30–32]. This strain is very common in Romania [33] and has also been isolated in Germany, Ireland, and Saudi Arabia [32, 34–37]. CC1-MRSA-IV is a traditional community acquired MRSA strain. This may suggest that the MRSA-positive tested person may have been colonized outside the hospital. Yet, recent observations suggest the spread CC1-MRSA-IV within and between hospitals and communities [37]. Thus, it is also possible that the acquisition of MRSA occurred within the hospital. The MRSA carrier in our study had contact to patients with MRSA within the last 4 weeks. It has been shown that work clothes, especially the gloves of HCW, are often contaminated with multidrug resistant bacteria during routine care [38, 39]. Unfortunately, we do not have data from patient admission screenings and patient MRSA status during the study period.

The response rate was rather low (31.3%). Sick leave, vacation and the regular distribution of working times may have prevented a certain proportion of the employees from having a chance to participate in the study. Study participation was on a voluntarily basis. Although individuals had the opportunity to participate anonymously, some may have declined participation due to a fear of adverse professional consequences, such as fear of stigmatization when tested positive for MRSA. A recent work by Peters and colleagues showed that German hospitals deal differently with MRSA-positive staff [19]. Recommendations concerning workers that are MRSA carriers range from following standard hygiene procedures to restricting MRSA-colonized workers from working with patients, or even requiring them to take time off from work. Moreover, in exceptional cases, it was reported that employees were fired from work due to permanent MRSA-colonization. Thus, the fear of stigmatization and job loss may have influenced study participation. There are recommendations for specific hygiene measures by the German Commission for Hospital Hygiene and Infection Prevention (KRINKO - Kommission für Krankenhaushygiene und Infektionsprävention) [29]. However, these recommendations concern work restrictions for MRSA positive staff during outbreaks. In non-outbreak situations, hospitals deal differently with MRSA colonized staff [19]. National regulations would be helpful for handling MRSA-colonized staff.

The major limitations of the study are the small sample size and the low response rate (149 of 575). However, participant characteristics matched general employee characteristics concerning age and sex suggesting low bias. Furthermore, we only tested staff members, and no patients were tested, making it difficult to make assumptions about the transmission paths [40]. Also, it would be useful to know whether the same MRSA strains are found in staff and patients. Another limitation is the cross-sectional study design which may have led to over- or underestimation of MRSA prevalence. Furthermore, we only obtained samples from the anterior nares. It has been shown that screening other body sites increases MRSA yield by about one third over nares alone [41]. Thus, subjects colonized with MRSA at other body sites (e.g. throat and axilla) may have been missed, leading to an underestimation of MRSA-prevalence.

## Conclusion

The results suggest a low prevalence of MRSA in a German cardiac care center in a non-outbreak setting. The results also correspond with the emerging trend of decreasing MRSA carriage prevalence in Europe that may be due to improved hygiene measures. Nevertheless, there is still a need for national regulations for dealing with MRSA-colonized staff in the healthcare sector.

## Abbreviations

HCW: Health care workers
MRSA: Methicillin Resistant *Staphylococcus aureus*
